# Treatment of Rheumatism

**Published:** 1876-06

**Authors:** Eug. C. Gehrung

**Affiliations:** Denver


### TREATMENT OF RHEUMATISM.

By EUG. C. GEHRUNG, M.D., Denver.

The Chairman of the Committee on Practice, mentioned in his report among "the late improvements," the beneficial effects obtained from the application of Plaster of Paris as an immovable dressing in acute articular rheumatism. To impress its importance more forcibly on the minds of the gentlemen present, I have concluded to give utterance to what little experience I have had with a similar mode of treatment, unconscious that others were experimenting in the same direction.

March 18, 1874, I was called to Miss K. S., suffering with acute articular rheumatism, spreading from joint to joint, first attacking the lower, then the upper extremities, and the chest. I concluded to bring into practice the plan I had decided upon by former reasoning, *i. e.*, to prevent change of temperature in the diseased joints, and, if possible, to prevent swelling. Consequently I wrapped the affected joint with a thick layer of cotton wool, and applied a roller bandage tightly from the toes to above the knee, which was, at the time, the only joint involved. The pain ceased almost immediately. I ordered an eighth of a grain of morphia at bedtime, to procure a good night's rest, and liberal nourishing diet. The next morning, the other knee, ankle and wrist, began to swell; bandages were applied with the same beneficial result, without removing the first, which, on account of the diminished swelling, had become moderately loose. To satisfy the patient, and partly myself, I ordered during the day, three doses, of two grains each, of propylamin, which was taken for about a week. Every night a sufficient dose of morphine was given to quiet the patient for the night. As pain in the chest was complained of, a broad bandage and cotton batting was applied around the chest also. On the twelfth day, I found my patient up and convalescing rapidly, have passed through acute articular rheumatism without having suffered pain of any consequence.

Several *subacute* cases I have since treated by the same plan, and with the same success.

A farmer called on me March 15, 1875, stating that his brother had suffered, for over three weeks, intolerable pains from acute rheumatism. I gave him directions as above, and he afterwards reported to me, "that the patient was almost immediately relieved from pain, and made rapid recovery."

Mrs. L. M., born during her mother's affliction with arthritis deformans, sent for me May 26, 1875. She was in a high fever, 104° f.; wrists, elbows and shoulder joints enlarged and painful. I repeated the same treatment as in the preceding cases; the propylamin was taken for three days only, and she progressed without much pain, although joint after joint became involved. On the third day the temperature was only 100°f. On my visit on Monday, 31st, or the sixth day of her illness, she was dressed, and stated that she had been up most all day yesterday, and that she was to-day completely free from pain or trouble of any kind.

To draw any conclusions from this small number of cases would be unwarrantable.

Whether the propylamin treatment was essential to the success, or whether there was something peculiar in the cases under observation—that they would have recovered as rapidly under any treatment—is a question which can be settled only by further experience—*Transactions Colorado Medical Society.*

